# Second-line ramucirumab therapy for advanced hepatocellular carcinoma (REACH): an East Asian and non-East Asian subgroup analysis

**DOI:** 10.18632/oncotarget.12780

**Published:** 2016-10-20

**Authors:** Joon Oh Park, Baek-Yeol Ryoo, Chia-Jui Yen, Masatoshi Kudo, Ling Yang, Paolo B. Abada, Rebecca Cheng, Mauro Orlando, Andrew X. Zhu, Takuji Okusaka

**Affiliations:** ^1^ Samsung Medical Center, Sungkyunkwan University School of Medicine, Seoul, Republic of Korea; ^2^ Asan Medical Center, University of Ulsan College of Medicine, Seoul, Republic of Korea; ^3^ National Cheng Kung University Hospital, Tainan City, Taiwan; ^4^ Kinki University School of Medicine, Osaka-Sayama City, Osaka, Japan; ^5^ Eli Lilly and Company, Bridgewater, NJ, USA; ^6^ Eli Lilly and Company, Indianapolis, IN, USA; ^7^ Eli Lilly and Company, Taipei, Taiwan; ^8^ Eli Lilly and Company, Buenos Aires, Argentina; ^9^ Massachusetts General Hospital Cancer Center, Harvard Medical School, Boston, MA, USA; ^10^ National Cancer Center Hospital, Tokyo, Japan

**Keywords:** Asians, alpha-fetoprotein, clinical trial, liver neoplasms, vascular endothelial growth factor receptor-2

## Abstract

**Purpose:**

REACH investigated second-line ramucirumab therapy for advanced hepatocellular carcinoma.

**Results:**

Median overall survival was 8.2 months for ramucirumab and 6.9 months for placebo (HR, 0.835; 95% CI, 0.634–1.100; *p* = 0.2046) for East Asians, and 10.1 months for ramucirumab and 8.0 months for placebo (HR, 0.895; 95% CI, 0.690–1.161; *p* = 0.4023) for non-East Asians. Median overall survival in patients with baseline alpha-fetoprotein ≥ 400 ng/mL was 7.8 months for ramucirumab and 4.2 months for placebo (HR, 0.749; 95% CI, 0.519–1.082; *p* = 0.1213) for East Asians (*n* = 139), and 8.2 months for ramucirumab and 4.5 months for placebo (HR, 0.579; 95% CI, 0.371–0.904; *p* = 0.0149) for non-East Asians (*n* = 111). The most common grade ≥ 3 treatment-emergent adverse events in East Asians and non-East Asians included hypertension and malignant neoplasm progression.

**Materials and methods:**

A post-hoc analysis of East Asians (*N* = 252) and non-East Asians (*N* = 313) in the intent-to-treat population was performed.

**Conclusions:**

In East Asians and non-East Asians, ramucirumab did not significantly prolong overall survival. In patients with baseline alpha-fetoprotein ≥ 400 ng/mL, a potentially larger survival benefit was observed in both subgroups. Safety for East Asians was similar to non-East Asians.

## INTRODUCTION

Among cancer deaths, liver cancer is the second most common cause [1]. Hepatocellular carcinoma represents approximately 70% to 90% of primary liver cancers [1, 2]. The incidence rates of liver cancer are highest in East Asian (EA) countries [1]. Intermediate rates occur in Southern Europe and Northern America, and the lowest rates occur in Northern Europe [1]. In general, EA patients have a poorer prognosis than non-EA patients. In the Asia-Pacific study of sorafenib versus placebo in EA patients, median overall survival (OS) in both treatment arms was shorter than the median OS in either arm of the SHARP study of sorafenib versus placebo in a global cohort of patients [3, 4]. Nonetheless, the relative hazard ratios for survival benefits were similar [3, 4]. Survival was also shorter for EA patients compared to non-EA patients in the GIDEON non-interventional study [5]. The reasons for the shorter survivals in EA patients remain unclear, but may include differences in tumor-related factors or patient characteristics. The most common cause of liver cancer in EA patients is hepatitis B virus infection, which is prevalent in this region, whereas hepatitis C virus or alcohol use are the most common causes of liver cancer in non-EA patients [6]. Disease management can also vary across regions [7], and EA patients are more likely to present at a more advanced stage of the disease [8, 9].

Vascular endothelial growth factor (VEGF) and VEGF receptor-2 (VEGFR-2)-mediated signaling are important in the proliferation of hepatocellular carcinoma tumors [10–13]. Ramucirumab, a recombinant human IgG1 monoclonal antibody, binds with high affinity and specificity to the extracellular domain of VEGFR-2, preventing angiogenesis via VEGF- and VEGFR-2-mediated signaling [14]. Ramucirumab in patients with advanced hepatocellular carcinoma as a second-line treatment following first-line therapy with sorafenib did not demonstrate a significant OS improvement over best supportive care (primary endpoint) in the phase III REACH trial [15]. However, improvements in progression-free survival (PFS) and response rate were observed [15]. In a pre-specified subgroup analysis of patients with a baseline alpha-fetoprotein (AFP) ≥ 400 ng/mL, ramucirumab treated patients had improved OS compared to placebo-treated patients [15].

A post-hoc subgroup analysis of the REACH trial was performed in advanced hepatocellular carcinoma EA and non-EA patients following first-line therapy with sorafenib to explore safety and efficacy of ramucirumab treatment in these patient populations.

## RESULTS

### Patients

**Figure 1:**
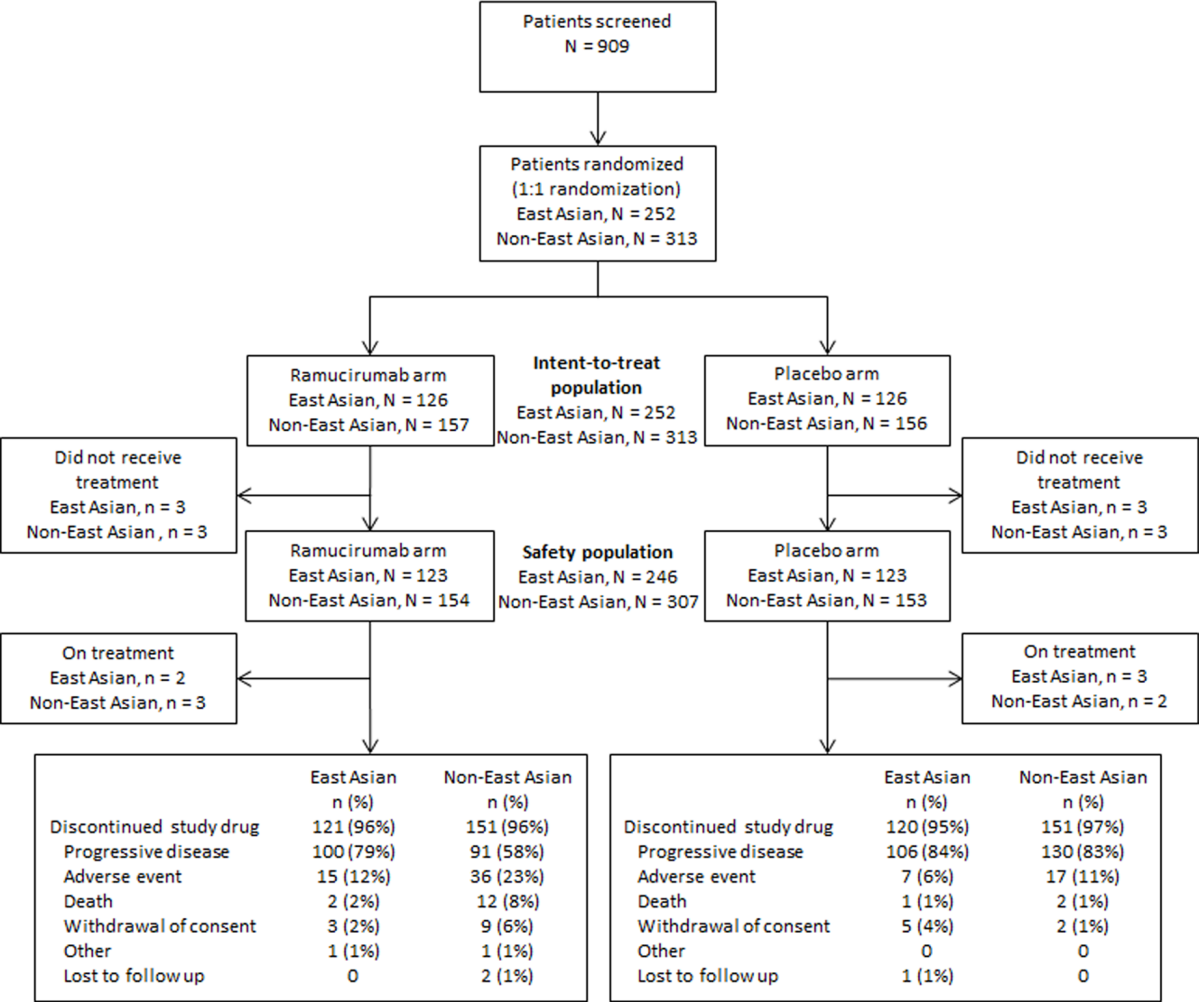
Trial profile for East Asian and non-East Asian patients

Figure 1 shows the CONSORT diagram for EA and non-EA patients. A total of 252 EA patients were randomized to receive ramucirumab (*N* = 126) or placebo (*N* = 126); 313 non-EA patients were randomized to receive ramucirumab (*N* = 157) or placebo (*N* = 156).

Baseline patient and disease characteristics for EA and non-EA patients were generally well balanced between treatment arms (Table 1). Differences between EA and non-EA patients were observed for age, Eastern Cooperative Oncology Group performance status (ECOG PS), etiology of liver disease, primary tumor present, presence of extra-hepatic spread, Barcelona Clinic Liver Cancer stage, baseline AFP, prior systemic therapy, and reasons for discontinuation of prior sorafenib therapy. Most differences were consistent with EA patients having a worse prognosis compared to non-EA patients.

**Table 1:** Baseline characteristics

	East Asian		Non-East Asian	
	Ramucirumab (*N* = 126)	Placebo (*N* = 126)	Ramucirumab (*N* = 157)	Placebo (*N* = 156)
Age, years				
Median (range)	61 (34–85)	59 (25–83)	66 (28–87)	64 (30–85)
< 65	80 (63.5)	83 (65.9)	70 (44.6)	79 (50.6)
≥ 65	46 (36.5)	43 (34.1)	87 (55.4)	77 (49.4)
Male	107 (84.9)	112 (88.9)	129 (82.2)	130 (83.3)
ECOG PS^a^				
0	63 (50.0)	63 (50.0)	96 (61.1)	90 (57.7)
1	63 (50.0)	63 (50.0)	61 (38.9)	66 (42.3)
Etiology of liver disease				
Hepatitis B	79 (62.7)	76 (60.3)	30 (19.1)	31 (19.9)
Hepatitis C	31 (24.6)	28 (22.2)	52 (33.1)	49 (31.4)
Significant alcohol use	10 (7.9)	13 (10.3)	49 (31.2)	50 (32.1)
Steatohepatitis (fatty liver)	3 (2.4)	4 (3.2)	16 (10.2)	16 (10.3)
Other	3 (2.4)	3 (2.4)	3 (1.9)	7 (4.5)
Unknown	10 (7.9)	8 (6.3)	30 (19.1)	26 (16.7)
Baseline Child-Pugh Class A	125 (99.2)	125 (99.2)	152 (96.8)	151 (96.8)
Primary tumor present	107 (84.9)	106 (84.1)	150 (95.5)	146 (93.6)
Macrovascular invasion present	40 (31.7)	37 (29.4)	42 (26.8)	42 (26.9)
Extrahepatic spread present	98 (77.8)	102 (81.0)	109 (69.4)	98 (62.8)
Baseline BCLC Stage				
Stage B	10 (7.9)	13 (10.3)	23 (14.6)	21 (13.5)
Stage C	116 (92.1)	113 (89.7)	134 (85.4)	135 (86.5)
Prior sorafenib therapy				
Sorafenib only	99 (78.6)	102 (81.0)	145 (92.4)	151 (96.8)
Sorafenib and other systemic therapy	27 (21.4)	24 (19.0)	12 (7.6)	5 (3.2)
Reason for discontinuation of sorafenib				
Progressive disease	116 (92.1)	112 (88.9)	130 (82.8)	127 (81.4)
Toxicity	10 (7.9)	14 (11.1)	27 (17.2)	29 (18.6)
Alpha fetoprotein				
< 400 ng/mL	60 (47.6)	53 (42.1)	100 (63.7)	97 (62.2)
≥ 400 ng/mL	66 (52.4)	73 (57.9)	53 (33.8)	58 (37.2)
Missing	0	0	4 (2.5)	1 (0.6)

Data are n (%) unless otherwise indicated.

a Performance status evaluated according to guidelines of the Eastern Cooperative Oncology Group (ECOG), with a performance status (PS) of 0 indicating asymptomatic, 1 restricted in strenuous activity but ambulatory and able to do light work, or 2 ambulatory and capable of all self-care but unable to work. Abbreviations: BCLC = Barcelona Clinic Liver Cancer staging system; ECOG PS = Eastern Cooperative Oncology Group performance status.

### Efficacy

**Figure 2:**
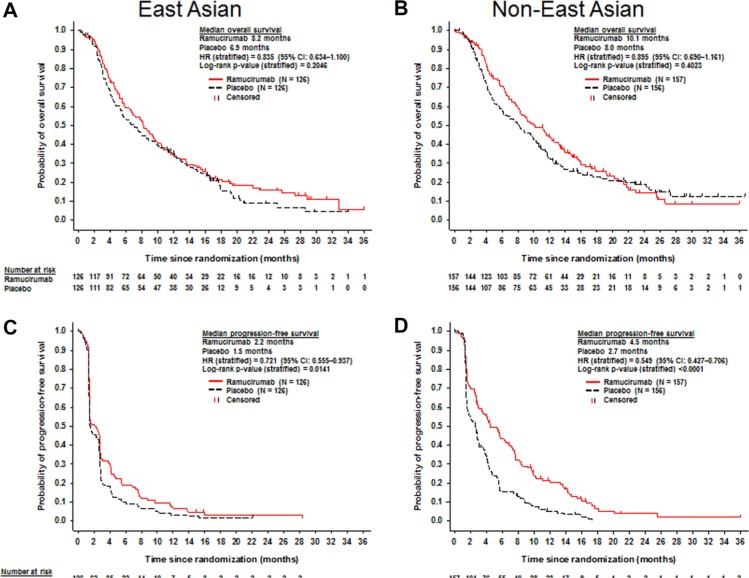
Kaplan-Meier plots of overall survival (A and B) and progression-free survival (C and D) for East Asian (A and C) and non-East Asian patients (B and D)

In EA patients, median OS for ramucirumab-treated patients was 8.2 months and 6.9 months for placebo-treated patients (stratified HR, 0.835; 95% CI, 0.634–1.100; *p* = 0.2046) (Figure 2). Median PFS was 2.2 months for the ramucirumab arm and 1.5 months for the placebo arm (stratified HR, 0.721; 95% CI, 0.555–0.937; *p* = 0.0141) (Figure 2). The objective response rate (ORR) was 5.6% (95% CI, 2.7–11.0) for the ramucirumab arm and 0.8% (95% CI, 0.1–4.4) for placebo arm (*p* = 0.0298) (Table 2). The disease control rate (DCR) was 47.6% for the ramucirumab arm and 42.1% for the placebo arm (*p* = 0.3568).

**Table 2:** Best overall response

	East Asian		Non-East Asian	
	Ramucirumab (*N* = 126)	Placebo (*N* = 126)	Ramucirumab (*N* = 157)	Placebo (*N* = 156)
Best overall response				
Complete response	0	0	1 (0.6)	0
Partial response	7 (5.6)	1 (0.8)	12 (7.6)	1 (0.6)
Stable disease	53 (42.1)	52 (41.3)	86 (54.8)	75 (48.1)
Progressive disease	57 (45.2)	62 (49.2)	40 (25.5)	67 (42.9)
Not evaluable or assessed	9 (7.1)	11 (8.7)	18 (11.5)	13 (8.3)
Objective response rate	7 (5.6)	1 (0.8)	13 (8.3)	1 (0.6)
95% CI	2.7–11.0	0.1–4.4	4.9–13.7	0.1–3.5
*p*-value	0.0298		0.0012	
Disease control rate^a^	60 (47.6)	53 (42.1)	99 (63.1)	76 (48.7)
95% CI	39.1–56.3	33.8–50.8	55.3–70.2	41.0–56.5
*p*-value	0.3568		0.0096	

Data are *n* (%) unless otherwise indicated.

a Denotes best response for complete response, partial response, or stable disease. Abbreviation: CI = confidence interval.

In non-EA patients, median OS for ramucirumab-treated patients was 10.1 months and 8.0 months for placebo-treated patients (stratified HR, 0.895; 95% CI, 0.690–1.161; *p* = 0.4023) (Figure 2). Median PFS was 4.5 months for the ramucirumab arm and 2.7 months for the placebo arm (stratified HR, 0.549; 95% CI, 0.427–0.706; *p* < 0.0001) (Figure 2). The ORR was 8.3% (95% CI, 4.9–13.7) for the ramucirumab arm and 0.6% (95% CI, 0.1–3.5) for the placebo arm (*p* = 0.0012) (Table 2). The DCR was 63.1% for the ramucirumab arm and 48.7% for the placebo arm (*p* = 0.0096) (Table 2).

**Figure 3:**
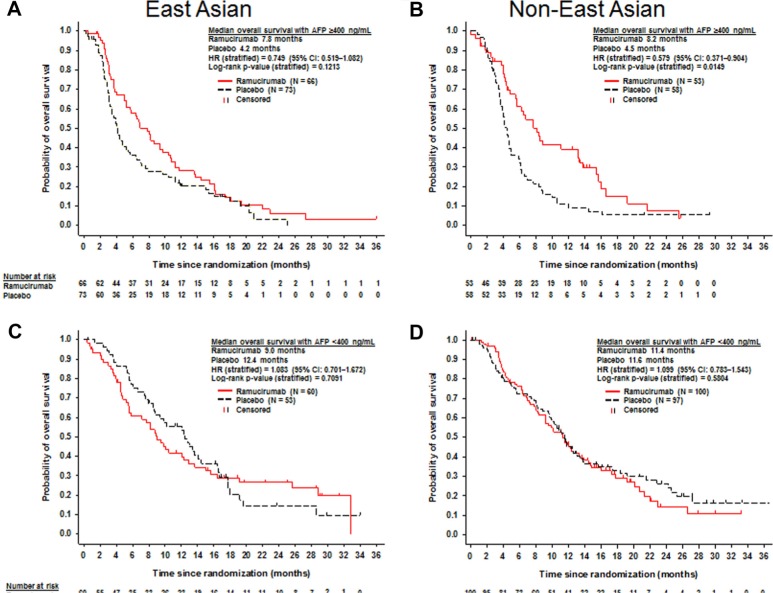
Kaplan-Meier plots of overall survival in patients with baseline alpha-fetoprotein ≥ 400 ng/mL (A and B) and alpha-fetoprotein < 400 ng/mL (C and D) for East Asian (A and C) and non-East Asian patients (B and D)

In EA patients with AFP ≥400 ng/mL (*n* = 139), median OS for the ramucirumab arm (*n* = 66) was 7.8 months and 4.2 months for the placebo arm (*n* = 73) (HR, 0.749; 95% CI, 0.519–1.082; *p* = 0.1213) (Figure 3). In non-EA patients with AFP ≥ 400 ng/mL (*n* = 111), median OS for ramucirumab-treated patients was 8.2 months (*n* = 53) and 4.5 months for placebo-treated patients (*n* = 58) (stratified HR, 0.579; 95% CI, 0.371–0.904; *p* = 0.0149) (Figure 3).

In EA patients with AFP < 400 ng/mL (*n* = 113), median OS for the ramucirumab arm (*n* = 60) was 9.0 months and 12.4 months for the placebo arm (*n* = 53) (HR, 1.083; 95% CI, 0.701–1.672; *p* = 0.7091) (Figure 3). In non-EA patients with AFP < 400 ng/mL (*n* = 197), median OS for ramucirumab–treated patients was 11.4 months (*n* = 100) and 11.6 months for placebo-treated patients (*n* = 97) (stratified HR, 1.099; 95% CI, 0.783–1.543; *p* = 0.5804) (Figure 3).

Post-discontinuation systemic anti-cancer therapies (PDT) were similar for ramucirumab and placebo-treated patients in both EA and non-EA subgroups; however, a higher percentage of EA patients received PDT than non-EA patients (EA: 37.3% for the ramucirumab arm vs. 38.1% for the placebo arm; non-EA: 20.4% for the ramucirumab arm vs. 26.9% for the placebo arm).

### Safety

The EA safety population consisted of 123 patients in the ramucirumab arm and 123 patients in the placebo arm. The non-EA safety population consisted of 154 patients in the ramucirumab arm and 153 patients in the placebo arm. The incidences of grade ≥ 3 treatment-emergent adverse events (TEAEs) were higher in the ramucirumab arm than the placebo arm for EA and non-EA patients (Tables 3 and 4). Any grade TEAEs occurring in at least 15% of patients and at a higher rate (at least 10% difference) in the ramucirumab arm than the placebo arm were peripheral edema, diarrhea, headache, thrombocytopenia, proteinuria, hypertension, hypoalbuminemia, and epistaxis for EA patients (Table 3), and peripheral edema, ascites, asthenia, hypertension, headache, and thrombocytopenia for non-EA patients (Table 4). Grade ≥ 3 TEAEs that occurred in at least 5% of patients and at a higher rate in the ramucirumab arm than the placebo arm were hypertension and malignant neoplasm progression for EA patients (Table 3; data not shown for malignant neoplasm progression), and hypertension, asthenia, ascites, general physical health deterioration, thrombocytopenia, and malignant neoplasm progression for non-EA patients (Table 4; data not shown for general physical health deterioration and malignant neoplasm progression).

**Table 3:** Adverse events in East Asian patients

	East Asian			
	Ramucirumab (*N* = 123)		Placebo (*N* = 123)	
	Any Grade	Grade ≥ 3	Any Grade	Grade ≥ 3
Treatment-emergent adverse events				
Any	119 (96.7)	64 (52.0)	110 (89.4)	48 (39.0)
Peripheral edema	41 (33.3)	0	15 (12.2)	0
Fatigue	28 (22.8)	2 (1.6)	20 (16.3)	4 (3.3)
Decreased appetite	26 (21.1)	2 (1.6)	26 (21.1)	0
Diarrhea	26 (21.1)	0	7 (5.7)	0
Headache	25 (20.3)	1 (0.8)	3 (2.4)	0
Ascites	24 (19.5)	4 (3.3)	14 (11.4)	4 (3.3)
Thrombocytopenia	24 (19.5)	5 (4.1)	6 (4.9)	0
Proteinuria	23 (18.7)	4 (3.3)	11 (8.9)	0
Pyrexia	22 (17.9)	0	14 (11.4)	0
Hypertension	21 (17.1)	8 (6.5)	8 (6.5)	1 (0.8)
Hypoalbuminemia	19 (15.4)	2 (1.6)	6 (4.9)	0
AAT increase	18 (14.6)	8 (6.5)	20 (16.3)	15 (12.2)
Epistaxis	18 (14.6)	0	5 (4.1)	0
Adverse events of special interest				
Liver injury/failure^a^	59 (48.0)	23 (18.7)	38 (30.9)	24 (19.5)
Bleeding/hemorrhage^a^	38 (30.9)	6 (4.9)	17 (13.8)	8 (6.5)
Gastrointestinal hemorrhage^b^	10 (8.1)	4 (3.3)	7 (5.7)	5 (4.1)
Pulmonary hemorrhage^b^	4 (3.3)	0	2 (1.6)	1 (0.8)
Hepatic hemorrhage^b^	1 (0.8)	1 (0.8)	2 (1.6)	2 (1.6)
Proteinuria^a^	24 (19.5)	4 (3.3)	11 (8.9)	0
Hypertension^a^	22 (17.9)	9 (7.3)	8 (6.5)	1 (0.8)
Renal failure^a^	10 (8.1)	2 (1.6)	6 (4.9)	0
Infusion-related reaction^a^	4 (3.3)	0	1 (0.8)	0
Arterial thromboembolic events^a^	1 (0.8)	0	2 (1.6)	1 (0.8)
Venous thromboembolic events^a^	1 (0.8)	0	1 (0.8)	1 (0.8)

Data are *n* (%). Only treatment-emergent adverse events in ≥ 15% of patients in the ramucirumab arm in East Asian patients are reported. A patient was only counted once for each category. Missing grades are counted in ‘Any Grade’. Adverse events were coded using MedDRA version 16.1.

a Pooled adverse event terms.

b Pooled adverse event category comprising synonymous MedDRA preferred terms. Abbreviation: AAT = aspartate aminotransferase.

**Table 4:** Adverse events in non-East Asian patients

	Non-East Asian			
	Ramucirumab (*N* = 154)		Placebo (*N* = 153)	
	Any Grade	Grade ≥ 3	Any Grade	Grade ≥ 3
Treatment-emergent adverse events				
Any	151 (98.1)	108 (70.1)	150 (98.0)	84 (54.9)
Peripheral edema	60 (39.0)	1 (0.6)	35 (22.9)	1 (0.7)
Ascites	50 (32.5)	9 (5.8)	26 (17.0)	7 (4.6)
Asthenia	48 (31.2)	13 (8.4)	33 (21.6)	4 (2.6)
Fatigue	36 (23.4)	4 (2.6)	38 (24.8)	4 (2.6)
Nausea	36 (23.4)	0	31 (20.3)	0
Decreased appetite	35 (22.7)	3 (1.9)	24 (15.7)	2 (1.3)
Hypertension	34 (22.1)	26 (16.9)	12 (7.8)	9 (5.9)
Abdominal pain	32 (20.8)	4 (2.6)	42 (27.5)	10 (6.5)
Headache	28 (18.2)	1 (0.6)	12 (7.8)	0
Cough	27 (17.5)	1 (0.6)	14 (9.2)	0
Diarrhea	25 (16.2)	3 (1.9)	31 (20.3)	1 (0.7)
Pyrexia	24 (15.6)	1 (0.6)	12 (7.8)	1 (0.7)
Thrombocytopenia	24 (15.6)	8 (5.2)	6 (3.9)	1 (0.7)
Constipation	23 (14.9)	0	26 (17.0)	0
Adverse events of special interest				
Liver injury/failure^a^	81 (52.6)	35 (22.7)	65 (42.5)	41 (26.8)
Bleeding/hemorrhage^a^	52 (33.8)	11 (7.1)	38 (24.8)	13 (8.5)
Gastrointestinal hemorrhage^b^	15 (9.7)	7 (4.5)	16 (10.5)	12 (7.8)
Pulmonary hemorrhage^b^	5 (3.2)	1 (0.6)	2 (1.3)	1 (0.7)
Hepatic hemorrhage^b^	1 (0.6)	1 (0.6)	0	0
Hypertension^a^	34 (22.1)	26 (16.9)	12 (7.8)	9 (5.9)
Proteinuria^a^	24 (15.6)	2 (1.3)	2 (1.3)	0
Infusion-related reaction^a^	16 (10.4)	3 (1.9)	1 (0.7)	0
Renal failure^a^	10 (6.5)	4 (2.6)	12 (7.8)	3 (2.0)
Venous thromboembolic events^a^	5 (3.3)	2 (1.3)	3 (2.0)	3 (2.0)
Arterial thromboembolic events^a^	1 (0.6)	0	2 (1.3)	0
Congestive heart failure^a^	0	0	2 (1.3)	1 (0.7)
Healing complication^a^	0	0	1 (0.7)	0

Data are *n* (%). Only treatment-emergent adverse events in ≥ 15% of patients in the ramucirumab arm in non-East Asian patients are reported. A patient was only counted once for each category. Missing grades are counted in ‘Any Grade’. Adverse events were coded using MedDRA version 16.1.

a Pooled adverse event terms.

b Pooled adverse event category comprising synonymous MedDRA preferred terms. Abbreviation: AAT = aspartate aminotransferase.

The incidences of adverse events of special interest (AESIs) are shown in Tables 3 and 4 for EA and non-EA patients, respectively. Any grade AESIs that were more common (at least 10% difference) in the ramucirumab arm than the placebo arm were liver injury/failure, bleeding/hemorrhage, proteinuria, and hypertension for EA patients (Table 3), and liver injury/failure, hypertension, and proteinuria for non-EA patients (Table 4). Grade ≥ 3 AESIs that occurred at a higher rate in the ramucirumab arm than the placebo arm were hypertension, proteinuria, and renal failure for EA patients (Table 3), and hypertension, renal failure, infusion-related reaction, and proteinuria for non-EA patients (Table 4).

## DISCUSSION

This subgroup analysis of REACH indicates that, while no significant OS benefit was shown in EA patients, there were improvements in PFS and ORR. Similar findings were noted in non-EA patients. Patients with AFP ≥400 ng/mL appeared to have a more favorable OS benefit in both the EA and non-EA groups, consistent with the findings in the overall intent-to-treat (ITT) population with AFP ≥ 400 ng/mL.

Overall, the survival benefit of ramucirumab was comparable in EA patients and non-EA patients, although EA patients had a shorter median OS compared with non-EA patients. A shorter median OS for EA patients versus non-EA patients was also observed in the SHARP and Asia-Pacific studies of sorafenib in hepatocellular carcinoma [3, 4]. The shorter median OS for EA patients in REACH may partly be due to a higher prevalence of baseline characteristics associated with poor prognosis in EA patients compared to non-EA patients. For instance, EA patients in REACH had a higher incidence of hepatitis B infection, macrovascular invasion, extrahepatic spread, Barcelona Clinic Liver Cancer stage C, increased concentration of AFP, and poorer ECOG PS than non-EA patients. In addition, more EA patients were aged less than 65 years compared with non-EA patients. Notably, patients in the Asia-Pacific study were also reported to have a higher incidence of hepatitis B, extrahepatic spread, Barcelona Clinic Liver Cancer stage C, poorer ECOG PS, and younger age compared to patients enrolled in the SHARP study [3, 4]. Post-discontinuation systemic anti-cancer therapy in REACH is unlikely to have contributed to the shorter OS in EA patients compared with non-EA patients given that a higher percentage of EA patients (38%) received PDT compared with non-EA patients (24%). Furthermore, no treatment has demonstrated a survival benefit to date in hepatocellular carcinoma after sorafenib treatment [15–18]. The improvement in PFS in EA patients was consistent with the improvement in non-EA patients, although PFS was shorter and the DCR was lower for EA patients than non-EA patients. The shorter PFS and DCR for EA patients may reflect the poorer prognosis and more rapidly progressive disease in the EA population. Despite known regional differences in the etiology and prognosis of hepatocellular carcinoma, ramucirumab demonstrated comparable survival efficacy in both EA and non-EA patients. These efficacy findings were similar to the overall REACH ITT population [15].

In patients with a baseline AFP ≥ 400 ng/mL, an improvement in OS was observed in both EA and non-EA patients treated with ramucirumab compared to placebo. This did not reach significance in the EA subgroup, likely due to the limitations of small sample size. Nonetheless, the difference in median OS was similar in the EA and non-EA subgroups and is generally consistent with the survival benefit observed in the overall ITT population with a baseline AFP ≥ 400 ng/mL in REACH [15]. This benefit was observed despite the overall poorer prognosis associated with EA patients compared to non-EA patients. We note that in the patients with baseline AFP ≥400 ng/mL, both EA and non-EA patients share a similar median OS in the placebo arm, which suggests that selection of this subset of patients may normalize any differences in prognosis between regions. Consistent with the overall ITT population with baseline AFP < 400 ng/mL [15], no OS benefit was observed in EA or non-EA patients with ramucirumab treatment. The OS results from these subgroup analyses demonstrate that a baseline AFP ≥ 400 ng/mL may identify patients who are most likely to benefit from ramucirumab treatment, regardless of whether they are from EA or non-EA regions.

The efficacy benefits for EA and non-EA patients were achieved with an acceptable safety profile. The majority of AESIs were grade 1–2, and the grade ≥ 3 AESIs were generally comparable between EA and non-EA patients. The observed safety profiles for EA and non-EA patients were consistent with the underlying disease state and the overall ITT populations in trials of ramucirumab [15, 19, 20]. Liver injury and bleeding are of particular concern in patients with advanced hepatocellular carcinoma, who often have underlying cirrhosis. In both EA and non-EA patients, increases in the low grade AESIs of liver injury/failure and bleeding/hemorrhage were observed in the ramucirumab arms compared with the placebo arms, but an increase in higher grade events was not observed. Notably, no increased rate of high grade AESIs was observed in EA patients compared to non-EA patients, despite the prevalence of poor prognostic characteristics in the EA subgroup that might have put these patients at higher risk with ramucirumab treatment.

This subgroup analysis has a number of limitations including the fact that the study was not designed or powered to show significance in the EA and non-EA subgroups, which makes it difficult to make accurate inferences. Furthermore, this analysis was post-hoc and caution should be used when interpreting the results. Despite these limitations, the efficacy observations in the subgroups defined by an AFP < or ≥ 400 ng/mL have been very consistent, and therefore seem unlikely to be due to chance.

In this subgroup analysis of the REACH trial, ramucirumab generally demonstrated consistent efficacy across EA and non-EA regions. The data indicate that patients with baseline AFP ≥ 400 ng/mL may be deriving the majority of the benefit observed in both EA and non-EA patients. Ramucirumab was well tolerated in both EA and non-EA patients. Further evaluation of ramucirumab in patients with advanced hepatocellular carcinoma is warranted. The REACH-2 trial will evaluate the efficacy and safety of ramucirumab in a global cohort of participants with hepatocellular carcinoma and elevated baseline AFP (ClinicalTrials.gov Identifier: NCT02435433).

## MATERIALS AND METHODS

### Study design and patients

The study design and demographic information for patients in REACH have been published previously [15]. Each center's institutional review board or independent ethics committee approved this study. The study followed the guiding principles of the Declaration of Helsinki and the Good Clinical Practice Guidelines of the International Conference on Harmonization. All patients provided written informed consent before enrollment. This study is registered with ClinicalTrials.gov, number NCT01140347.

### Randomization and procedures

Randomization and procedures have been published previously [15]. Patients were randomly assigned in a 1:1 ratio to receive either ramucirumab 8 mg/kg or placebo intravenously every two weeks until disease progression, unacceptable toxicity, or withdrawal of consent. All patients received best supportive care. Predefined dose modifications were allowed to manage treatment-related toxicity. Randomization was stratified by geographic region (region 1 [*n* = 65]: Brazil, Canada, and the United States vs. region 2 [*n* = 248]: Australia, Europe, and Israel vs. region 3 [*n* = 252]: East Asia) and etiology of liver disease (hepatitis B vs. hepatitis C vs. other etiologies). Region 1 consisted of Brazil (*n* = 27), Canada (*n* = 1), and the United States (*n* = 37); region 2 consisted of Australia (*n* = 11), Austria (*n* = 8), Belgium (*n* = 8), Bulgaria (*n* = 6), the Czech Republic (*n* = 20), Finland (*n* = 3), France (*n* = 59), Germany (*n* = 40), Hungary (*n* = 1), Israel (*n* = 2), Italy (*n* = 51), the Netherlands (*n* = 3), Norway (*n* = 2), Portugal (*n* = 2), Romania (*n* = 7), Spain (*n* = 21), Sweden (*n* = 2), and Switzerland (*n* = 2); and region 3 consisted of Hong Kong (*n* = 24), Japan (*n* = 93), Philippines (*n* = 1), South Korea (*n* = 70), Taiwan (*n* = 58), and Thailand (*n* = 6).

### Statistical analysis

Statistical methodology was the same as published previously [15]. The EA and non-EA subgroups were separately analyzed. The EA patient population was defined and analyzed as patients enrolled at study sites in region 3. The non-EA patient population was defined and analyzed as patients enrolled at study sites in regions 1 and 2 combined.
